# The newly emerged COVID-19 disease: a systemic review

**DOI:** 10.1186/s12985-020-01363-5

**Published:** 2020-07-08

**Authors:** Endeshaw Chekol Abebe, Tadesse Asmamaw Dejenie, Mestet Yibeltal Shiferaw, Tabarak Malik

**Affiliations:** 1 Department of Biochemistry, College of Health Sciences, Debre Tabor University, Debre Tabor, Ethiopia; 2 Department of Biochemistry, College of Medicine and Health Sciences, University of Gondar, Gondar, Ethiopia; 3 Department of Medicine, College of Health Sciences, Debre Tabor University, Debre Tabor, Ethiopia

**Keywords:** COVID-19, *SARS-CoV-2*, Outbreak, Pandemic

## Abstract

Coronaviruses are large family-RNA viruses that belong to the order *Nidovirales*, family *Coronaviridae*, subfamily *Coronavirinae*. The novel COVID-19 infection, caused by a beta coronavirus called *SARS-CoV-2*, is a new outbreak that has been emerged in Wuhan, China in December 2019. The most common symptoms of COVID-19 are fever, cough, and dyspnea. As per the March 12, 2020, WHO report, more than 125,048 confirmed COVID-19 cases and over 4613 deaths have been identified in more than 117 countries. It is now regarded as a pandemic that seriously spread and attack the world. The primary means of transmission is person to person through droplets that occurred during coughing or sneezing, through personal contact (shaking hands), or by touching contaminated objects. So far, there is no effective therapy and vaccine available against this novel virus and therefore, only supportive care is used as the mainstay of management of patients with COVID-19. The mortality rate of COVID-19 is considerable. This work aimed to provide insight on the newly emerged COVID-19, in the hope to gain a better understanding on the general overview, epidemiology, transmission, clinical features, diagnosis, treatment, and clinical outcomes as well as the prevention and control of COVID-19.

## Background

Coronavirus disease (COVID-19) is an illness caused by a novel coronavirus now called *severe acute respiratory syndrome coronavirus 2*. It was first identified amid an outbreak of respiratory illness cases in Wuhan City, Hubei Province, China. Initially, it was reported to the WHO on December 31, 2019. On January 30, 2020, the WHO declared the COVID-19 outbreak a global health emergency. WHO declared COVID-19 a global pandemic on March 11, 2020. This work aimed to provide insight on the newly emerged COVID-19, in the hope to gain a better understanding on the general overview, epidemiology, transmission, clinical features, diagnosis, treatment, and clinical outcomes as well as the prevention and control of COVID-19.

## Main text

Coronaviruses (CoV) are a large family of pleomorphic, crown-shaped, enveloped positive sense single-stranded RNA viruses that belong to the order *Nidovirales*, family *Coronaviridae*, subfamily *Coronavirinae*. There are hundreds of coronaviruses, most of which are zoonotic that are present in humans and various animals (including pigs, camels, bats, and cats). They are classified into four genera RNA containing viruses pathogenic for mammals, namely *alpha-, beta-, gamma-, and delta-coronavirus*. So far only the first two coronaviruses are identified in humans and the latter two have only been identified in animals, with bats being host to the largest genomic variety [1–3].

The CoV causes an illness with a wide range of clinical features that usually result in mild to moderate upper-respiratory tract illnesses, like the common cold and in some cases causing the more severe type of illness involving the respiratory, gastrointestinal, hepatic and neurologic systems. Seven coronaviruses species are known to cause human disease, of which four prototypic CoV cause endemic and epidemic respiratory diseases, including the *alpha-coronaviruses-229E* and *-NL63*, and the *beta-coronaviruses OC43* and *Human coronavirus HKU1*. Due to the rapid mutation and genetic recombination of CoV, three new CoV have emerged since the 1960s. These new viruses can have more serious outcomes in people, and responsible for coronavirus outbreaks for three times in the past two decades in the twenty-first century, which have emerged from animal reservoirs to cause severe disease and global concerns. Those diseases are SARS (severe acute respiratory syndrome) which is caused by *SARS-CoV* (*beta-coronavirus*), and emerged as an outbreak in late 2002; MERS (Middle East respiratory syndrome) which is caused by *MERS-CoV* (*beta-coronavirus*) and it is an ongoing outbreak emerged in 2012; and the newly emerged COVID-19 [4–6].

**Fig. 1:**
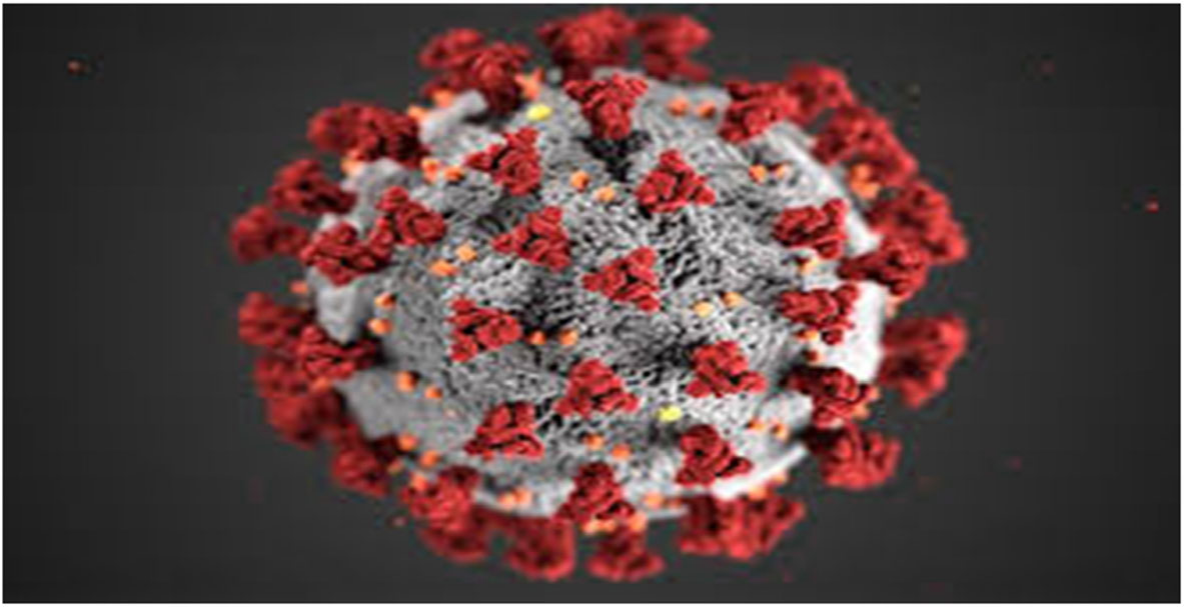
Structure of novel coronavirus (SARS-CoV-2) (CREDIT: CDC/ Alissa Eckert, MS; Dan Higgins, MAM)

The novel coronavirus disease 2019 (COVID-19) emerged in the Huanan Seafood Market, where livestock animals are also traded in China on December 30, 2019, and becoming a global issue to control its spread. It was initially observed in Wuhan city of China as a cluster of patients with pneumonia of unknown etiology and reported to the World Health Organization (WHO) China bureau in Beijing. A week later, on January 7, 2020, a new coronavirus that has not been previously identified in humans, was isolated from these patients by the Chinese scientists [7]. This newly recognized strain of coronaviruses that have been found to be the etiologic agent of COVID-19 was called *severe acute respiratory syndrome coronavirus 2 (SARS-CoV-2*). Like *SARS-CoV* and *MERS-CoV*, this novel CoV is classified under the group of *beta-coronavirus* (Fig. 1). It was initially referred to as novel coronavirus 2019 (2019-nCoV) but was given the official name of COVID-19 by WHO on February 11, 2020 [8].

Similar to the SARS epidemic, this outbreak has occurred during china’s annual Lunar New Year holiday, which is the most famous traditional festival in China, during which nearly 3 billion people travel from all sides of the country. These conditions favoured the transmission of this highly contagious disease and become severe problems in the prevention and control of the outbreak [9, 10].

## Epidemiology

**Fig. 2:**
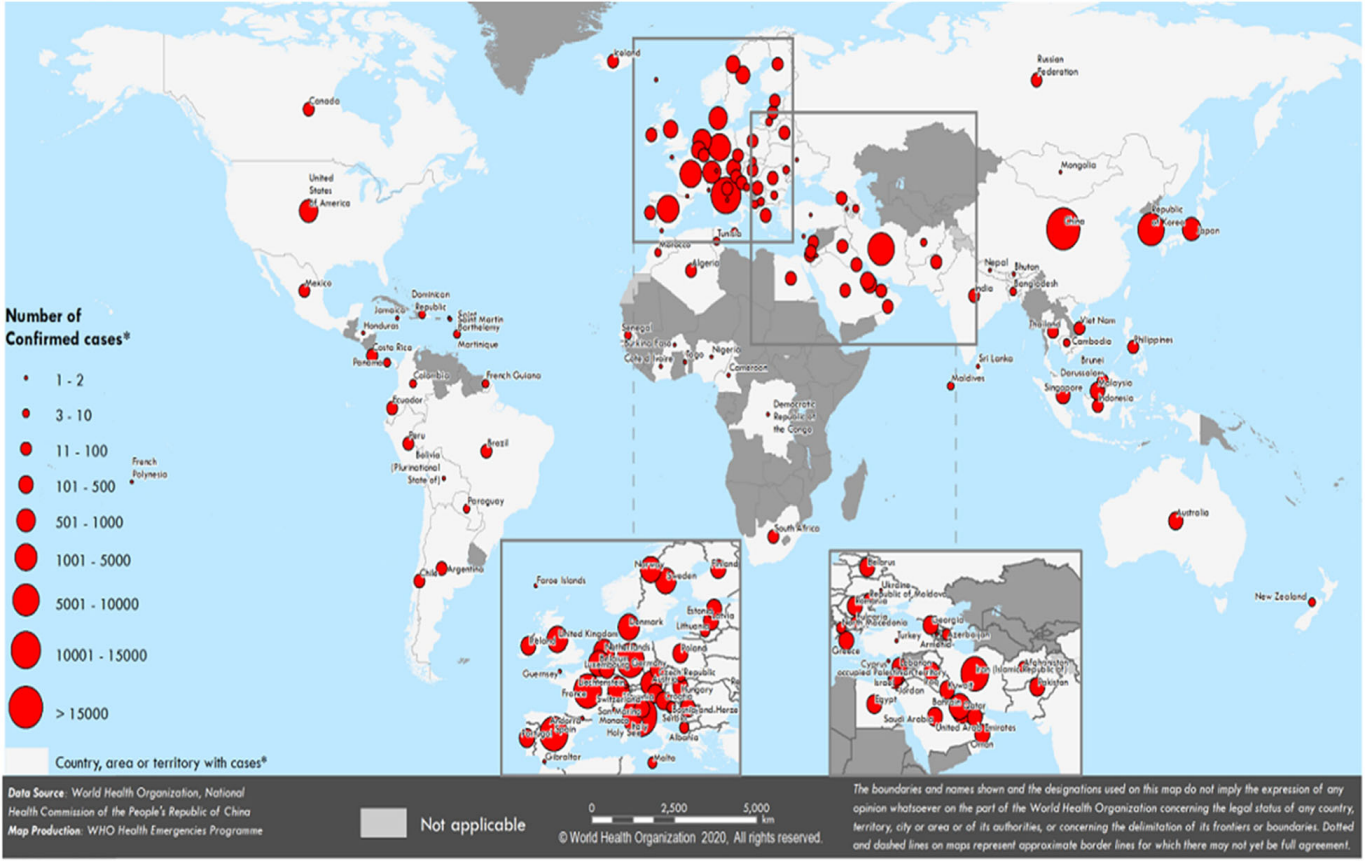
Distribution of reported confirmed COVID-19 cases by Countries, territories or areas as of 12 March 2020 [12]

**Fig. 3:**
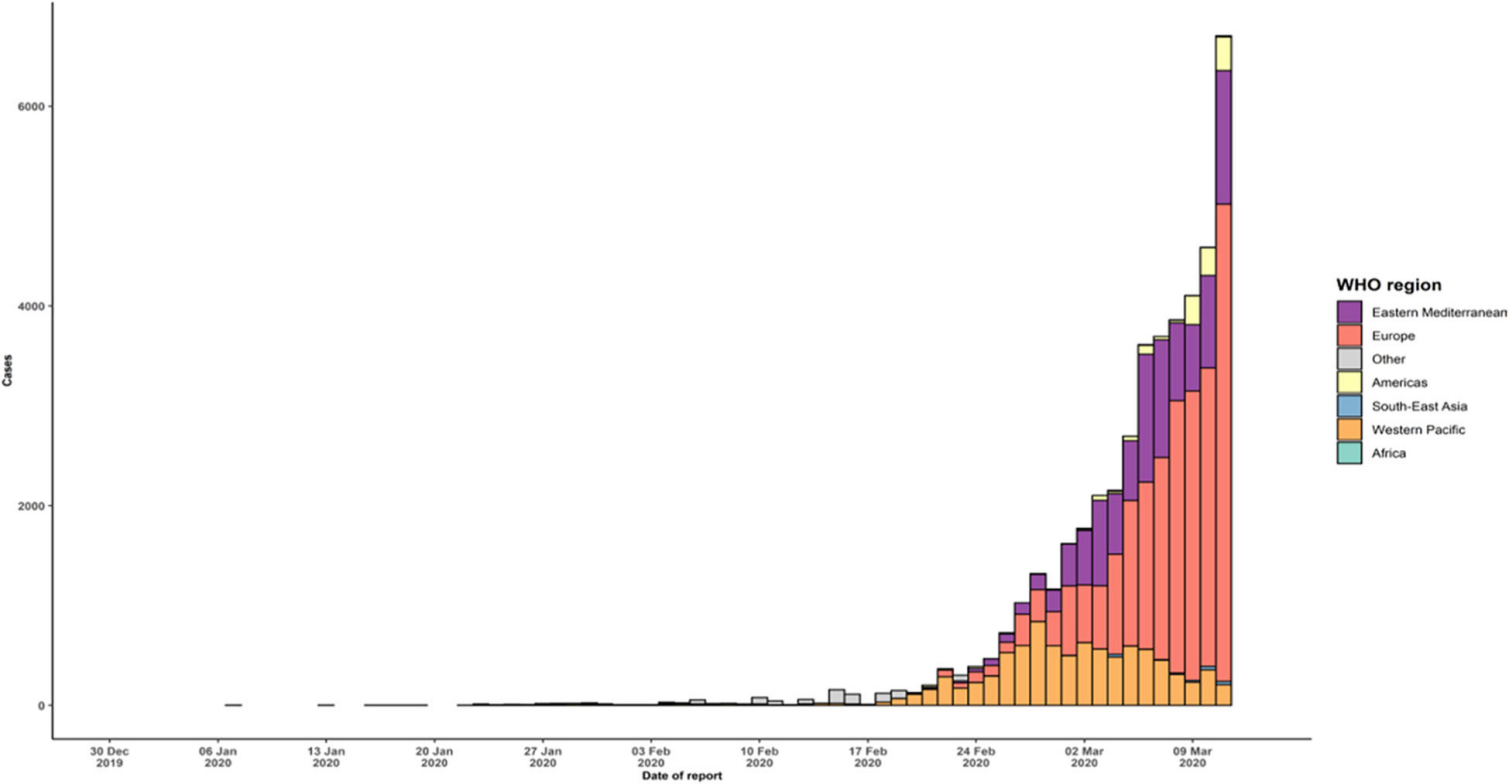
Epidemic curve of confirmed COVID-19 cases reported outside of China (*n* = 44,067), by date of report and WHO region through 12 March 2020 [12]

The current novel coronavirus disease 2019 (COVID-19) that causes pneumonia is a highly infectious disease, and the ongoing outbreak has affected a huge part of populations around the world. It has been declared by WHO as a global public health emergency of international concern on January 30, 2020 [11]. It is now spread rapidly from its first site of outbreak, Wuhan City, throughout Hubei province to other provinces in China and around the world. The epidemic is now reported to expand to over 117 countries as per March 12, 2020, WHO report. Since the start of the outbreak, more than 125,048 confirmed COVID-19 cases have been identified and over 4613 deaths have been globally reported. Of these, the majority (64.8%) cases were detected in China while the rest (35.2%) were identified outside china. The global distribution of COVID-19 cases as of March 12, 2020 WHO situation reports 52 is shown in Fig. 2. The number of countries reporting confirmed COVID-19 cases is now increasing from time to time according to WHO daily report. The epidemic curve of confirmed COVID-19 cases outside China is depicted in Fig. 3 [12].

The total number of COVID-19 cases is likely to be higher due to the inherent difficulties in identifying and counting mild and asymptomatic cases, especially in those developing countries having poor settings for case detection. Besides, the still-insufficient testing capacity for COVID-19, which means many suspected and clinically diagnosed cases are not yet counted in the denominator [13]. Furthermore, COVID-19 infected more people than either of its two predecessors, SARS and MERS. SARS which was emerged and declared by WHO as an outbreak in November 2002 in Guangdong Province of China and contained on July 5, 2003, with a total of 8096 SARS cases and 774 deaths across 29 countries while MERS emerged in 2012 in Saudi Arabia and is still ongoing outbreak and is thus far responsible for 2494 confirmed cases and 858 deaths across 27 countries [9, 10].

The novel COVID-19 more commonly affected males, and the median age range of patients is 49 to 59 years. Nearly all reported cases have occurred in adults (median age 59 years). Generally, older people are twice as likely to have serious COVID-19 illness. Just over 2% of cases were under 18 years of age, of which fewer than 3% developed severe or critical diseases [8, 10, 14]..

Based on the study done on 72,314 cases in China by Wu and McGoogan, the overall case-fatality rate (CFR) of COVID-19 was 2.3%. The CFR was higher in those cases aged 80 years and above (8.0%) and among critical COVID-19 cases (49.0%). The CFR was also higher among those with preexisting comorbid conditions:10.5% for cardiovascular disease, 7.3% for diabetes, 6.3% for chronic respiratory disease, 6.0% for hypertension, and 5.6% for cancer [10]. Despite much higher CFRs for SARS (9.6%) and MERS (34.4%), COVID-19 has led to more total deaths due to the large number of cases [15].

## Modes of transmission

Research evidences suggest *SARS-CoV-2*, like *SARS-CoV* and *MERS-CoV*, is zoonotic that was transmitted to from animals. It has been declared that *SARS-CoV-2* is originated from wild bats**.** However, a considerable transmission of COVID-19 is occurring among people who are in close contact with one another (within about 6 ft) and also through droplets produced when an infected person coughs or sneezes. These droplets can be inhaled into the lungs or drops into the nearby mouths or noses of people [16]. This human-to-human transmission of COVID-19 is confirmed by a recent report from many infected healthcare workers in Wuhan. In addition, it may be possible that a person can get COVID-19 by touching the virus-infected inanimate objects and having contact with their own mouth, nose, or possibly their eyes unwashed, but this is not thought to be the main way the virus spreads. The most symptomatic (the sickest) the people are, the most contagious they thought to be. But some spread might be possible with this new coronavirus during the asymptomatic phase though this is not thought to be the main way the virus spreads [17, 18].

The transmission also occurs in hospital settings though it is not the major means of COVID spread. This is supported from the study by Wang and his colleagues in which COVID-19 affected health professionals, 40 (29%) and hospitalized patients, 17 (12.3%) and hospital-acquired transmission was suggested as the presumed mechanism of infection [19]. Hospital-acquired infection occurred in 13.5% of patients as per a report of Yang et al [20].

Moreover, the virus that causes COVID-19 seems to show a “community spread” in which the virus is spreading easily and sustainably in the community and the people have been infected without noticing how or where they contacted with the infection and scientists are still trying to determine how *SARS-CoV-2* spread to people [18, 21].

As per the findings from a small-scale study by Chen and his colleagues it has been reported that COVID-19 is not transmitted vertically from infected pregnant women to their fetus. However, this needs further investigation to surely arrive at any conclusion. The study also reported that there is currently no evidence for intrauterine infection caused by vertical transmission in women who develop COVID-19 pneumonia in late pregnancy [11].

The epidemic curves reflect as it may be a mixed outbreak pattern, with early cases suggestive of a continuous common source, potentially zoonotic spillover at Huanan Seafood Wholesale Market, and later cases suggestive of a propagated source as the virus began to be transmitted from person to person. Thus, COVID-19 rapidly spread from a single city to the entire country of China in just 30 days, which quickly overwhelmed the health and public health services of China, particularly in Wuhan City and Hubei Province [22]. Several factors have been forwarded for the rapid spread of this new virus: one is Wuhan is the capital of China’s Hubei province, with more than 11million inhabitants, and it is a major hub of transportation, which increases person-to-person contact and increases the chance of exporting cases to other locations. The unavailability of effective antiviral therapy and vaccine makes the disease difficult to prevent and control the spread. As a result, the outbreak spread to many Chinese provinces, and then to the Asian continent. The first non-Chinese case of the infection was reported from Thailand on January 13, 2020. The source of infection was from a Chinese tourist who has traveled to Thailand. Other cases are then continued to be reported in many countries of the world [7, 16, 17].

## Clinical features of COVID-19

**Fig. 4:**
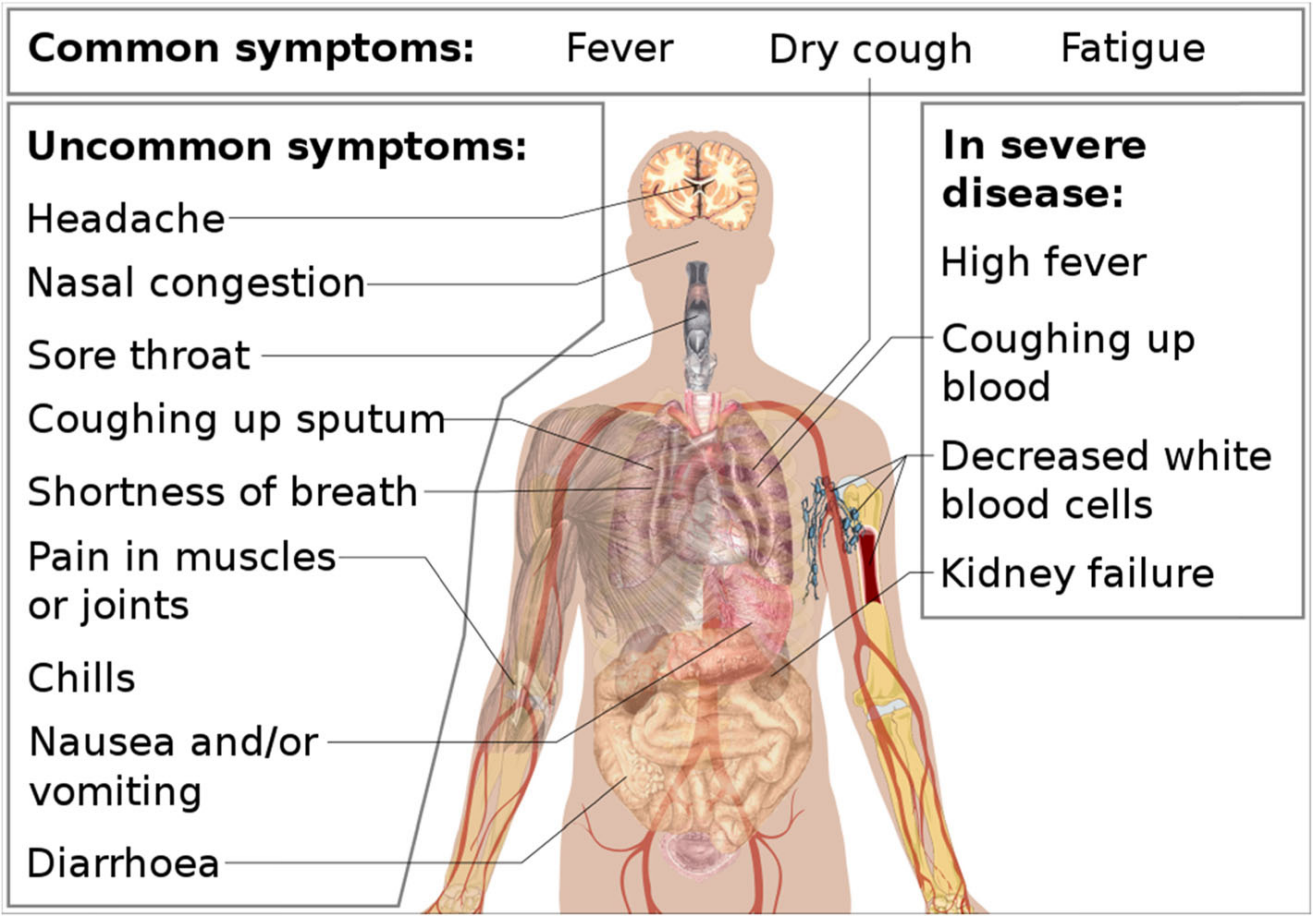
Symptoms of COVID-19 (source: Mikael Häggström, M.D)

More than 80% of COVID-19 illnesses are asymptomatic or can be symptomatic with mild symptoms such as respiratory symptoms, fever, cough, shortness of breath, and breathing difficulties. In some patients, COVID-19 results in severe illness with serious complications: severe pneumonia, acute respiratory distress syndrome (ARDS), acute respiratory failure, pulmonary edema, sepsis, septic shock or multiple organ failure, and even death [12]. Symptoms may appear 2–14 days after exposure. The median duration from onset of symptoms to radiological confirmation of pneumonia was 5 days while the median duration from onset of symptoms to ICU admission was 9.5 days [20]. The most common and frequently reported symptoms of COVID-19 are fever (98%), cough (77%), and dyspnea (63.5%) whereas less common symptoms are myalgia, malaise, arthralgia, chest pain, nasal congestion, runny nose, headache, sore throat, and diarrhea (Fig. 4) [11, 14, 20]. But not all these symptoms are present in COVID-19 cases. For instance, among 52 critically ill patients, six (11%) did not experienced fever until 2–8 days after the onset of symptoms related to *SARS-CoV-2* infection [20]. COVID-19 infected pregnant women have been reported to have a similar pattern of clinical characteristics as the non-pregnant adult patients [11].

## Diagnosis of COVID-19

Clinical diagnosis of COVID-19 can be made based on symptoms, exposures, and chest imaging. Even though COVID-19 manifest with different symptoms, none of these symptoms are present in every patient and there are no specific signs or symptoms that could suggest COVID-19 compared to symptoms and signs of respiratory illnesses caused by other viruses, such as influenza and common cold [21, 23]. Therefore, a thorough and meticulous clinical examination needs to be done before arriving at the final diagnosis of COVID-19 (Table 1). Besides, a COVID-19 suspected individual needs to be evaluated and confirmed for *SARS-CoV-2* using viral nucleic acid test called quantitative real time-PCR (qRT-PCR) using respiratory tract samples (e.g, throat swabs). Samples from amniotic fluid, cord blood, and neonatal throat swab can also be used for the diagnosis of *SARS-CoV-2* using PCR in pregnant women [11]. Moreover, chest CT might have a high diagnostic value because of its typical images of virus infection, high accuracy with a low false-negative rate, and time efficiency. Therefore, in addition to using qRT-PCR which is the gold standard for the diagnosis of COVID-19 pneumonia, it is beneficial to do relevant clinical examinations including complete blood counts (CBC), inflammatory markers and chest radiography and a comprehensive evaluation of a patient’s medical history, epidemiological exposure, and symptoms [8, 11].

**Table 1:**
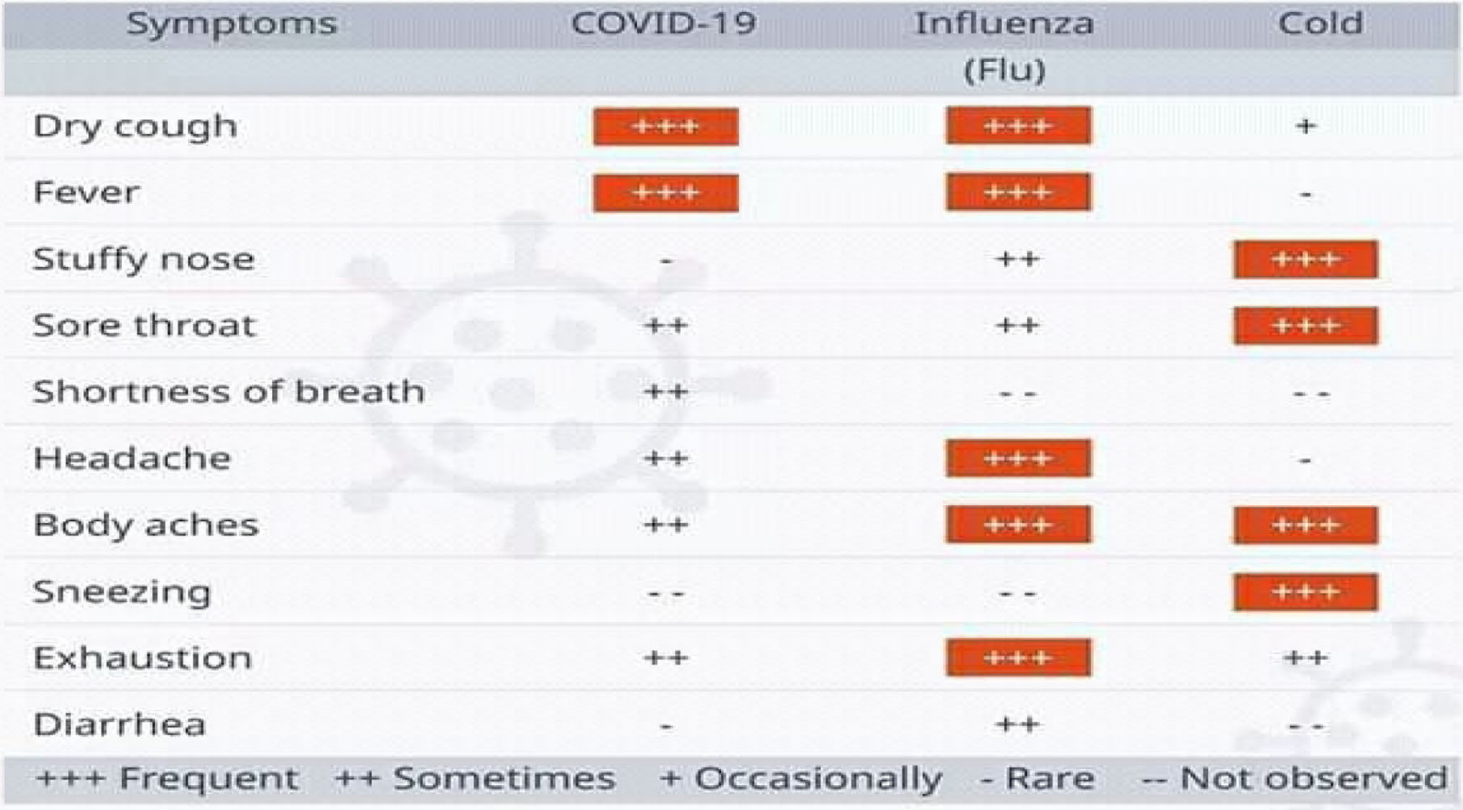
Symptoms of COVID-19 compared to influenza and common cold [12, 21].

From the CBC, lymphopenia (63%) appears as a prominent and the most likely occurring laboratory finding along with leukopenia (9–25%), leukocytosis (24–30%), and thrombocytopenia (12%). The increased concentrations of transaminases (ALT or AST) are also the laboratory findings of COVID-19, in which elevated transaminases were noted in 37% of whom extreme elevations are rare. Inflammatory markers such as CRP and ESR are elevated (68 and 84% respectively) while procalcitonin is normal in most cases. Chest X-ray and CT findings show bilateral infiltrates and unilateral involvement in 75 and 25%, respectively [8, 11, 14].

## Case definitions of COVID-19

For easy reference and surveillance, case definitions of COVID-19 are set by WHO based on the currently available informations [12] and defines COVID-19 case as:

### Suspect case

i. If a patient with a severe acute respiratory infection (fever, cough, and requiring admission to hospital), and with no other etiology that fully explains the clinical presentation and a history of travel to or residence in a country/area or territory reporting local transmission during the 14 days prior to symptom onset, OR
ii. A patient with an acute respiratory illness AND having been in contact with a confirmed or probable COVID-19 case in the last 14 days prior to the onset of symptoms OR
iii. A patient with a severe acute respiratory infection (fever and at least one sign/symptom of respiratory disease (e.g., cough, shortness breath) AND requiring hospitalization and with no other etiology that fully explains the clinical presentation.

### Probable case

- If a suspect case for whom testing for COVID-19 is inconclusive. Inconclusive being the result of the test reported by the laboratory. OR
- A suspect case for whom testing could not be performed for any reason.

### Confirmed case

- If a person with laboratory confirmation of 2019-nCoV infection, irrespective of clinical signs and symptoms.

## Treatment and clinical outcomes of COVID-19

Patient supportive care is typically the mainstay of treatment of COVID-19 as there are no specific effective antiviral therapies that have been identified so far. Standard supportive management including care to relieve symptoms and advanced vital organ support are indicated for respiratory disease and complications [10, 23]. Mechanical ventilation is the main supportive treatment for critically ill patients, which involves invasive methods such as endotracheal intubation and noninvasive method like supplementary oxygen therapy (target SpO2 ≥ 90% in non-pregnant adults and SpO2 ≥ 92–95% in pregnant patients) [20]. In addition, other supportive cares that should be given for severely ill patients involve close monitoring for signs of deterioration, conservative fluid management, giving antipyretics and /or analgesics, administering appropriate empirical therapy immediately (within an hour) of sepsis identification, and managing sepsis and septic shock by following sepsis guideline [23].

Without sufficient evidence, patients were given intravenous glucocorticoids and antiviral agents. Intravenous glucocorticoids were commonly used in patients with SARS and MERS pneumonia though their efficacy remains controversial and their use to treat COVID-19 infection is also controversial. Hence, systemic corticosteroids should not be routinely given for the treatment of viral pneumonia or ARDS outside of clinical trials unless they are indicated for another reason: septic shock or other disease processes. Currently, in the USA, Remdesivir, which was tested against Ebola, and Kaletra (lopinavir and ritonavir combination) are suggested to be the potential treatments against the novel coronavirus. Favorable outcome was brought with the intravenous remdesivir treatment of the first COVID-19 case in the USA. But clinical trials are underway to come up with solid evidence to recommend these drugs to routinely prescribe for COVID-19 and thus, the ongoing clinical trials might shed some light on the safety and efficacy of these drugs as treatment [14, 20, 24].

At this time, it is difficult to predict the mortality of COVID-19 as it has been fluctuating due to the source of information. But the WHO estimated the overall CFR between 2 and 3% [12]. However, the mortality of critically ill patients with COVID-19 pneumonia is considerable. According to a single-centered, retrospective study by Yang and his colleagues, the survival time of the non-survivors is likely to be within 1–2 weeks after ICU admission. For instance, of those critical patients requiring mechanical ventilation, 81% had died by 28 days. Moreover, the poor clinical outcome of COVID-19 patients was reported to be associated with older age and underlying comorbidities**.** Older patients (> 65 years) with comorbidities and ARDS are at increased risk of death [20].

## Prevention and control of COVID-19

The high mutation rate, different means of transmission of the novel virus as well as unavailability of the vaccine against COVID-19 makes it difficult to control the spread. Developing a vaccine is underway and hopes to begin a phase 1 trial within 3 months. As a consequence, countries are now focusing on traditional public health outbreak response tactics such as isolation, quarantine, social distancing, and community containment as targeted antiviral drugs and vaccines are not yet available for COVID-19 [21, 25].

The best way to prevent illness is to promptly implement infection control measures to avoid being exposed to this virus. Infection control measures are integral parts of the clinical management of patients and should be initiated at the point of patient’s admission to the hospital. Standard precautions should always be routinely applied in all areas of health care facilities. The WHO advised the world the shift from containment to mitigation would be wrong and dangerous as COVID-19 is a controllable pandemic. So, we can control or contain the person-to-person spread of COVID-19 infection by doing the following standard precautions/recommendations [12, 23].

- Regular hand-washing often with soap and water for at least 20 s, especially after going to the bathroom; before eating; and after blowing your nose, coughing, or sneezing. If soap and water are not available, use an alcohol-based hand sanitizer containing at least 60% alcohol. Always wash hands with soap and water if hands are visibly dirty.
- Avoid touching your eyes, nose, or mouth with unwashed hands.
- Avoid close contact with anyone showing symptoms of respiratory illness such as coughing and sneezing.
- Stay home when you are sick.
- Covering mouth and nose with a tissue when coughing and sneezing, then throw the tissue in the trash.
- Thoroughly cooking meat and eggs.
- Clean and disinfect frequently touched equipment, objects, and surfaces using a regular household cleaning spray or wipe.
- Prevention of needle-stick or sharps injury
- Safe waste management and environmental cleaning
- Use of personnel protective equipment (PPE) to avoid direct contact with patients’ blood, body fluids, secretions (including respiratory secretions), and non-intact skin.
- Following Center for disease control  (CDC) recommendation for facemask: CDC recommends facemasks to those people who show symptoms of COVID-19 to help prevent the spread of the disease to others. Otherwise, CDC does not recommend that healthy people wear a facemask to protect themselves from COVID-19.
- People who think they may have been exposed to COVID-19 should contact their healthcare provider immediately to be treated immediately and to prevent the spread [12, 21].

## Conclusion

In conclusion, the novel COVID-19 infection is a new outbreak that has been emerged in Wuhan, China in December 2019. It is caused by a novel *beta-coronavirus*, resulting from genetic recombination, called *SARS-CoV-2*, The most common symptoms of COVID-19 are fever, cough, and dyspnea. As per the March 12, 2020 WHO report, more than 125,048 confirmed COVID-19 cases and over 4613 deaths have been identified in 117 countries. It is now regarded as a pandemic that seriously spread and attack the world. The primary means of transmission is person to person through droplets that occurred during coughing or sneezing, through personal contact (shaking hands), or by touching contaminated objects. The overall CFR is between 2 and 3%. This figure is however higher in the elderly and those with underlying health conditions**.** Older patients (> 65 years) with comorbidities and ARDS are at increased risk of death. So far, there is no effective therapy and vaccine against this novel virus and therefore, only supportive care is used as the mainstay of clinical management of patients with COVID-19. Infection prevention and control measures are integral parts of the clinical management and are the best option we currently have for containment of COVID-19 spread.

## Data Availability

Not applicable.

## Abbreviations

ALT: Alanine Amino Transaminase
ARDS: Acute Respiratory Distress Syndrome
AST: Aspartate Amino Transaminase
CBC: Complete Blood Count
CFR: Case-Fatality Rate
*CoV*: *Coronaviruses*
COVID-19: Coronavirus Disease 2019
CT scan: Computed Tomography Scan
ICU: Intensive Care Unit
*MERS-CoV*: *Middle East Respiratory Syndrome Coronaviruses*
PCR: Polymerase Chain Reaction
RNA: Ribonucleic Acid
*SARS-CoV-2*: *Severe Acute Respiratory Syndrome Coronavirus-2*
USA: United States of America
WHO: World Health Organization
